# The Total Antioxidant Capacity and the Total Phenolic Content of Rice Using Water as a Solvent

**DOI:** 10.1155/2021/5268584

**Published:** 2021-03-27

**Authors:** C. Priyanthi, R. Sivakanesan

**Affiliations:** ^1^Faculty of Technology, University of Jaffna, Sri Lanka; ^2^Faculty of Medicine, University of Peradeniya, Sri Lanka

## Abstract

**Background:**

The present study evaluates the antioxidant properties of some Sri Lankan red rice varieties using water extracts.

**Methods:**

Water extracts of rice varieties Attakkari, Bg2907, and Bg407 were used in this study. The total antioxidant capacity was measured by ferric reducing antioxidant power (FRAP), 2,2-diphenyl-1-picrylhydrazyl (DPPH) radical scavenging, and reducing power assays. The total phenolic content (TPC), total flavonoid content (TFC), monomeric anthocyanin, and condensed tannin contents were measured by Folin-Ciocalteu, aluminium chloride, pH differential, and vanillin assays, respectively.

**Results:**

It was observed that mean FRAP, DPPH, reducing power, TPC, TFC, monomeric anthocyanin content, and condensed tannin content were in the range of 0.561 ± 0.113 to 0.695 ± 0.077 mmol/100 g fresh weight (FW), 26.07 ± 3.08 to 53.66 ± 7.61 mg/mL FW, 33.49 ± 4.105.14 to 40.81 ± 3.65 mg/mL, 0.676 ± 0.078 to 0.900 ± 0.057 mg tannic acid equivalent (TAE)/g, 5.36 ± 0.75 to 6.38 ± 0.82 mg TAE/g FW, 0.0202 ± 0.005 to 0.0292 ± 0.009 mg/g FW, and 0.078 ± 0.015 to 0.104 ± 0.017 mg TAE/g FW, respectively. Significant differences were observed in DPPH, reducing power, and TPC among rice varieties (*p* < 0.05). Rice variety Attakkari had the highest total antioxidant capacity (TAC), scavenging activity, reducing power, TPC, TFC, monomeric anthocyanin content, and condensed tannin content followed by Bg2907 and Bg406.

**Conclusion:**

Total phenolic compounds, total flavonoid, and condensed tannin are the major antioxidants in all three varieties of rice while the monomeric anthocyanin is only a minor antioxidant.

## 1. Background

In Sri Lanka, rice is the staple food and there are over 300 different traditional rice varieties. Rice plays an important role in supplying energy and nutrients in Sri Lankan's life. In addition to macronutrients, rice contains antioxidant activity compounds such as phenolic acids, flavonoids, anthocyanins, proanthocyanidins, tocopherols, tocotrienols, *γ*-oryzanol, and phytic acid. The phenolic compounds in rice are the phenolic acids, flavonoids, and tannins. The biological effects of rice mainly include antioxidant activity, anti-inflammation, anticancer, and antidiabetic activities [1]. The polyphenolic compounds found in abundance in whole grains are phenolic acids and flavonoids [2]. Phenolic compounds are bioactive substances widely distributed in plants. However, antioxidant capacity of each material differs due to different amounts of these compounds. The antioxidant compounds are recognized to have protective functions against oxidative damage and associated with reduced risk of chronic diseases [3].

Solid-to-liquid extraction is the most common method used to recover natural antioxidants from plant materials. Different organic solvents including methanol, ethanol, acetone, hexane, ethyl acetate, and mixtures of methanol, ethanol, or acetone with water are used for extraction. Extraction rate may vary with different types of solvents used. In order to avoid structural changes to the target antioxidant compounds during extraction, the types and concentrations of organic solvents would be carefully selected. Many studies have been focused on antioxidant activity of rice using an organic solvent. However, limited antioxidant activity studies have been conducted on Sri Lankan rice varieties using water as a solvent.

Hence, a study was conducted to estimate total phenolic content (TPC), total flavonoid content (TFC), total anthocyanin content (TAC), condensed tannins, 2,2-diphenyl-1-picrylhydrazyl (DPPH) radical scavenging activity, reducing power assay, and ferric ion reducing antioxidant power (FRAP) in some selected Sri Lankan varieties. In this study, water is used as an extraction medium as to its edibility and to avoid the undesirability of organic solvent on volatile organic compounds to environment. Water is the safest and also most environmentally friendly and easily obtainable solvent. It is also significantly less expensive than organic solvents, which have been traditionally used for plant bioactive extractions.

## 2. Materials

### 2.1. Chemicals and Instruments

Acetic acid, sodium acetate, 2,4,6-tripyridyl-s-triazine (TPTZ), ferric chloride hexa-hydrate, ferrous sulphate, 2,2-diphenyl-1-picrylhydrazyl (DPPH), methanol, sodium phosphate, potassium ferricyanide, trichloroacetic acid, Folin-Ciocalteu's phenol reagent, gallic acid, tannic acid, anhydrous sodium carbonate, aluminium chloride, catechin, sodium hydroxide, sodium nitrite, potassium chloride, vanillin, and hydrochloric acid were obtained from Sigma Chemicals Company (MO, USA). All the chemicals were of analytical grade, and distilled water was used throughout. Centrifugation was done using a centrifuge (MSE, UK), and a digital weighing scale (PA 313, Ohaus, USA) was used in weighing operations. Absorbance was measured with a UV-Vis spectrophotometer (UV 1800, Shimadzu, Japan).

## 3. Methods

### 3.1. Extraction of Rice

Rice grains were dehusked manually, and rice was obtained. One gram of each dehusked rice sample was transferred into a motor and ground into fine particles. Then, they were extracted with 10 mL of distilled water for 5 min. The water extracts were centrifuged at 5000 × *g* for 15 min to obtain clear supernatant. This 0.1 g/mL centrifuged extract was used for FRAP, reducing power, DPPH, total anthocyanin content, and condensed tannins. The extract was further diluted for total polyphenol content and total flavonoid content. Two millilitres of the extract was mixed with 1 mL of distilled water.

### 3.2. Determination of Ferric Reducing Antioxidant Power (FRAP)

The FRAP assay is based on the reduction of the Fe(III)-TPTZ complex to the ferrous form at low pH. This reduction was monitored by measuring the absorption change at 593 nm [4]. One millilitre of the working reagent was mixed with 20 *μ*L of the extract, and the absorbance at 593 nm was recorded exactly after 4 min of incubation at room temperature. The absorption of 1000 *μ*M ferrous sulphate standard was also measured. FRAP values are expressed as mmol of Fe(II) equivalent per 100 g rice.

### 3.3. Determination of 2,2-Diphenyl-1-picrylhydrazyl (DPPH) Radical Scavenging Ability

DPPH radical scavenging activity of the rice extract was determined according to the method reported [5]. A series of extracts (10, 20, 30, and 40 mg/mL) were prepared using distilled water, and the total volume was maintained as 1 mL. Two hundred and fifty microlitres of 0.5 mM methanolic solution of DPPH was added and vortexed. Test tubes were incubated for 30 min in the dark at room temperature. The absorbance was measured at 517 nm. Distilled water was used as a control.

The scavenging activity of the rice extract was expressed as 50% inhibition concentration (IC_50_) (mg/mL) and was obtained by interpolation (concentration vs. % free radical scavenging activity) from linear regression analysis.

### 3.4. Determination of Ferric Reducing Power

The method described by Yen and Duh [6] was used. Different concentration series of rice extracts were prepared, and the total volume of the solution was maintained as 400 *μ*L. Each sample was mixed with 1 mL of phosphate buffer (0.3 M, pH 6.6) and 1 mL of 1% K_3_[Fe(CN)_6_] and incubated at 50°C for 20 min. Then, 1 mL of 10% trichloroacetic acid was added. Two millilitres of the solution was mixed with 2 mL distilled water and 0.4 mL of 1% FeCl_3_·6H_2_O, and absorbance was measured at 700 nm after 30 min.

The reducing power of the rice extract was expressed as 0.5 absorbance concentration, and EC_50_ (mg/mL) was obtained by interpolation (concentration vs. absorbance) from linear regression analysis.

### 3.5. Determination of Total Phenolic Content (TPC)

The TPC of extracts was determined using a Folin-Ciocalteu reagent [7]. Fifty microlitres of the extract was added to 0.5 mL Folin-Ciocalteu reagent and vortexed. Four hundred microlitres of sodium carbonate solution (75 g/L) was added to the mixture after 3 min reaction time. Thereafter, the mixture was vortexed thoroughly. The absorbance of the resulting blue color was measured at 765 nm against a blank after 30 min of incubation at room temperature. All samples and readings were prepared and measured in triplicate. Tannic acid was used as the standard, and TPC was expressed as mg tannic acid (TA) equivalent per 100 g rice.

### 3.6. Determination of Total Flavonoid Content

Total flavonoid contents of each sample were determined using the colorimetric method [8]. Briefly, 250 *μ*L of extract solution was diluted with 1.25 mL distilled water and mixed with 75 *μ*L of 5% NaNO_2_. After 5 min, 150 *μ*L of 10% AlCl_3_ was added and then incubated for 6 min and 500 *μ*L of 1 mol/L NaOH was subsequently added. The absorbance was measured immediately at 510 nm. Tannic acid was used as the standard, and TPC was expressed as mg tannic acid (TA) equivalent per 100 g rice.

### 3.7. Determination of Total Anthocyanin Content

Anthocyanin content was estimated by a pH differential method [9]. Two dilutions of rice extracts were prepared: one with 0.025 M potassium chloride buffer (pH 1.0) and the other extract with 0.4 M sodium acetate buffer (pH 4.5) diluting each with previously determined dilution factor (extract: buffer 1 : 4 (*v*/*v*)). Diluted extracts were measured at 510 and 700 nm, respectively, after 20 min equilibrium time. The content of total anthocyanin was calculated using the following formula. Absorbance (Abs) was measured at *λ*_max_ nm (500 nm) and at 700 nm. Anthocyanin concentration was calculated and expressed as cyanidin-3-glycoside equivalent (mg/g FW).

(1) $$\begin{array}{c} \text{Anthocyanin contents }\left(\text{cyanidin}‐3‐\text{glucoside equivalents},\text{mg}/\text{L}\right)=\frac{\left[\left(A\times \text{MW}\times \text{DF}\times 1000\right)\right]}{\varepsilon \times 1},\\ A=\left(A535-A700\right)\text{pH}1.0-{\left(A535-A700\right)}_{\text{pH}4.5}, \end{array}$$

where MW is 449.2 g/mol for cyanidin-3-glucoside, *ε* is the 26,900 molar extinction coefficient, in L mol^–1^ cm^–1^, for cyd-3-glu, 1 is the path length in cm, and 1000 is the factor for conversion from g to mg. MW is the molecular weight, DF is the dilution factor, and *ε* is the molar absorptivity. It was converted to mg of total anthocyanin content/100 g sample.

### 3.8. Determination of Condensed Tannins

Condensed tannins were determined by the vanillin-HCl method of [10]. Two hundred microlitres of the extract was pipetted out into a test tube. Then, 1.5 mL of 4% vanillin in methanol and 750 *μ*L of concentrated HCl were added and vortexed. Tubes were incubated for 15 min. The absorbance was measured at 500 nm. Catechin (0.1 g/L) was used as the standard, and condensed tannins were expressed as mg catechin equivalent per 100 g rice.

### 3.9. Statistical Analysis

Rice samples were analysed five times in duplicate, and the mean values, standard deviations (SD), and correlation values (*r*^2^) were calculated. Statistical analysis was conducted on data using ANOVA and the general linear model, with SAS System version 9.1 for Windows. Mean separations were examined using the *t*-test. The differences were considered significant at *p* < 0.05.

## 4. Results and Discussion

### 4.1. Ferric Reducing Antioxidant Power

The total antioxidant capacity describes the ability of different food antioxidants in scavenging preformed free radicals which has been suggested as a tool for investigating the health effects of antioxidant-rich foods.

As shown in Table 1, sample Attakkari had the highest total antioxidant capacity (TAC) of 0.70 ± 0.077 mmol/100 g FW. Samples Bg2907 and Bg406 had total antioxidant capacity of 0.57 ± 0.075 and 0.56 ± 0.113 mmol/100 g FW, respectively. There were no significant differences among these three varieties at *p* < 0.05 as these three rice samples are red rice. Studies of [11] showed the mean FRAP value of red rice ranging from 0.9 to 8.1 mmol Fe(II)/100 g of DW. In the same study, they reported the mean FRAP value of Sri Lankan red rice ranging from 0.9 to 2.4 mmol Fe(II)/100 g of DW. Another latest study by Samaranayake et al. [12] showed that the mean FRAP value of traditional Sri Lankan rice varieties ranged from 2.47 to 6.79 mmol FeSO_4_/100 g bran. In traditional Sri Lankan varieties, mean FRAP values ranged from 8.30 to 11.02 mmol FeSO_4_/100 g bran suggesting the possibility of using Sri Lankan traditional rice bran as a viable source of antioxidant for nutraceuticals and functional foods [13]. These values were higher than those in the present study since water was used as an extraction medium. This suggests that ethanol is more effective in the extraction of antioxidants than water. High amounts of antioxidants are concentrated in the rice bran.

Studies conducted on antioxidant properties of traditional rice varieties of Sri Lanka have shown that red rice had significantly high antioxidant properties compared to white rice [13, 14]. Sorghum exhibited the highest antioxidant activity at 20.92 ± 2.69 mg/g Trolox equivalent in the FRAP assay [15].

### 4.2. DPPH Radical Scavenging Activity

The results were stated as the concentration of the extract to inhibit 50% of DPPH radical (IC_50_). The absorption decreases when antioxidants give protons to the free radical. The lower value of IC_50_ indicates a higher antioxidant value. As shown in Table 1, rice Attakkari showed the highest scavenging activity with the IC_50_ value of 26.07 ± 3.08 mg/ml, followed by Bg2907 (32.66 ± 7.45 mg/ml) and Bg406 (53.66 ± 7.61 mg/ml). As the lowest IC_50_ value corresponds to the highest antioxidant activity, sample Attakkari contained significantly higher (*p* < 0.05) antioxidants than rice Bg406. These results were higher than the antioxidant activity of sorghum where the highest antioxidant activity was recorded as 21.02 ± 5.17 mg/g Trolox equivalent [15].

In a previous study, Dutta et al. [16] observed 6.01-14.47 mg/mL as the IC_50_ value for Bangladesh varieties which is lower than our value. Genetic variations among the rice varieties and use of methanol as the extraction medium could play a vital role in this discrepancy.

### 4.3. Reducing Power

The reducing power is also an indicator of antioxidant activity. As shown in Table 1, Attakkari had the lowest EC_50_ value of 33.49 ± 4.10 mg/mL FW which indicates the highest reducing power. This was followed by the reducing power of Bg2907 (38.55 ± 3.52 mg/mL FW). The reducing power of Bg406 (40.81 ± 3.65 mg/mL of FW) was significantly lower (*p* < 0.05) compared to that of Attakkari and Bg2907.

Sompong et al. [11] found weak DPPH activity in Sri Lankan rice variety compared to Thai and Chinese rice. Marimuthu et al. [17] observed 31-154.66 mg/g of reducing power activity in brown rice.

### 4.4. Total Phenolic Content (TPC)

The results are expressed as tannic acid equivalents (mg TAE/g FW) and are shown in Table 2. Significant difference was observed between rice Attakkari and other two varieties Bg2907 and Bg406. The possible reason for this variation could be due to several factors including difference in the rice variety, seasonal variation, and soil condition [18]. No significant difference was observed in total phenolic content between Bg2907 and Bg406 varieties.

The highest phenolic content of 0.900 ± 0.057 mg TAE/g FW was observed in Attakkari. This was followed by Bg406 with 0.742 ± 0.077 mg TAE/g FW. The lowest phenolic content of 0.676 ± 0.078 mg TAE/g FW was obtained for Bg2907 which was significantly lower (*p* < 0.05) than Attakkari.

The results of the present study corroborate with the findings of Basu et al. [19] which reported the mean phenolic content ranging from 0.6 to 0.9 mg TAE/g for unpolished rice varieties. In another study by Sompong et al. [11], they observed large variations in total phenolic acids between red rice and black rice grown at different locations in Sri Lanka. They reported that TPC of three different Sri Lankan red rice ranged from 0.79 to 2.08 mg FAE/g DW basis. These values were expressed as ferulic acid equivalent. In a study by Dutta et al. [16], they reported the TPC of Bangladesh brown rice ranging from 0.14 to 0.25 mg gallic acid equivalent (GAE)/g which is lower than that in the present study. In a study by Yu et al. [20], TPC in wild Chinese rice was recorded as 1.42 to 5.3 mg GAE/g. The variation could be due to the polishing of rice, cultivar varieties, rice color, and extraction solvent. Ferulic acid and protocatechuic acid are the major constituents in red rice [11]. Rice bran is the richest source of phenolic antioxidants in rice grains [14]. Removal of rice bran during polishing could considerably reduce the antioxidant activity of red rice. Phenolic compounds were major antioxidant constituents in cereals, medicinal herbs, vegetables, fruits, and spices. Total phenolic content observed in this study is much higher than the values for white rice, black rice, brown rice, mung bean, foxtail millet, proso millet, barley, sorghum, and adlay [21]. In a study by Adom and Liu [22], the total phenolic content of rice was 5.56 *μ*mol/g of grain which was lower than those of corn, wheat, and oats.

### 4.5. Total Flavonoid Content (TFC)

The results for TFC are given in Table 2 as tannic acid equivalents (mg TAE/g FW). Total flavonoid contents of the rice Attakkari, Bg2907, and Bg406 were 6.38 ± 0.82, 5.59 ± 0.76, and 5.36 ± 0.75 mg TAE/g FW, respectively. There were no significant differences among the varieties.

The reported flavonoid contents in the studies were 6.60 to 12.80 mg/g rutin equivalent, which was higher than what we obtained in the present study [18]. In another recent study in Chinese wild rice, there was 2.26 to 4.35 mg catechin/g [20]. The total flavonoid content of unpolished indica rice variety was 46.8-52.1 mg tannic acid equivalent/g and reduced to 11–13.6 mg tannic acid equivalent/g in polished rice [19]. This difference could be due to the expression of results as different equivalents and different genotypes, environmental conditions, or methanol as the extraction medium.

There are a variety of flavonoids that have been identified in rice: flavanols (flavan-3-ols), flavanones, flavanonols, flavones, flavonols, and isoflavones, which generally occur as O- or C-glycosides. The flavonoid found mainly is tricin (77%), and other flavonoids are luteolin (14%), apigenin (6%), quercetin (3%), isorhamnetin (1%), myricetin (<1%), and kaempferol (<1%) [23].

### 4.6. Monomeric Anthocyanin Content

Anthocyanins are one subclass of flavonoids and are responsible for most of the red and purple colors of fruits, vegetables, flowers, and other plant tissues or products. Black and red rice varieties have cyanidin 3-glucoside and peonidin 3-glucoside as the main anthocyanin compounds. These phytochemical compounds usually accumulated in pericarp or bran of rice kernels [24]. Anthocyanins are the primary functional components in many varieties of rice especially pigmented rice [1]. Anthocyanins have been reported to have strong antioxidant capacity and health-beneficial potentials such as anti-inflammatory disease, anticancer, anticardiovascular disease, and obesity prevention [1].

According to Table 2, the monomeric anthocyanin contents of the rice Attakkari, Bg2907, and Bg406 were 0.0292 ± 0.009, 0.0247 ± 0.004, and 0.0202 ± 0.005 mg/g FW, respectively. No significant differences were observed among the varieties. Sompong et al. [11] found total anthocyanin to be between 0.0033 and 0.0091 mg/g in Sri Lankan red rice varieties where polished rice was used as samples. The observed variation may be due to different rates of polishing. The outer layer of the rice is removed during polishing which reduces the anthocyanin contents and further affects the total polyphenol and antioxidant activity.

### 4.7. Condensed Tannin Content (CTC)

The condensed tannin contents of the rice Attakkari, Bg2907, and Bg406 were 0.104 ± 0.017, 0.099 ± 0.023, and 0.078 ± 0.015 CE/g FW, respectively (Table 3). No significant differences were observed among the varieties.

### 4.8. Correlation between Antioxidant Capacity, Total Flavonoid, Total Phenolic, Monomeric Anthocyanin, and Condensed Tannin Content

Table 4 compares the correlation (*r*^2^) between TAC of rice varieties and individual antioxidants. A strong positive correlation between the TAC and total phenolic content was observed in Attakkari, Bg2907, and Bg406. This indicates that phenolic compounds could be the main component responsible for total antioxidant activity of rice.

Moreover, a strong positive correlation between the total phenolic content and total flavonoid content indicates that the flavonoids are the major polyphenols in the three rice varieties. Also, there was a strong positive correlation between the total flavonoid and monomeric anthocyanin content, indicating that monomeric anthocyanin could be one of the major flavonoids in rice. The strong positive relationship between the TAC and the monomeric anthocyanin suggests that contribution of monomeric anthocyanin to the TAC might be high. However, there was a weak positive relationship between condensed tannins and total phenolic content. Thus, condensed tannins might not be among the main polyphenols of rice.

Overall, the TAC measured as FRAP, IC_50_, and EC_50_ value showed a strong positive correlation with the total phenol and total flavonoid as well as the monomeric anthocyanin content of all three rice varieties indicating the significance of those phenolic compounds contributing to TAC. A weak positive correlation between condensed tannins and TAC shows that condensed tannins have a minor result on TAC of these three rice varieties.

## 5. Conclusions

This study concludes that rice variety Attakkari shows higher antioxidant content than Bg2907 and Bg406. Mean FRAP, DPPH, reducing power, TPC, TFC, monomeric anthocyanin content, and condensed tannin content are in the range of 0.561 ± 0.113 to 0.695 ± 0.077 mmol/100 g FW, 26.07 ± 3.08 to 53.66 ± 7.61 mg/mL FW, 33.49 ± 4.105.14 to 40.81 ± 3.65 mg/mL, 0.676 ± 0.078 to 0.900 ± 0.057 mg TAE/g, 5.36 ± 0.75 to 6.38 ± 0.82 mg TAE/g FW, 0.0202 ± 0.005 to 0.0292 ± 0.009 mg/g FW, and 0.078 ± 0.015 to 0.104 ± 0.017 mg CE/g FW, respectively. Total phenolic compounds, total flavonoid, and condensed tannin are the major antioxidants in all three varieties of rice while the monomeric anthocyanin is only a minor antioxidant.

## Figures and Tables

**Table 1:** Total antioxidant capacities measured by ferric reducing antioxidant power, DPPH radical scavenging capacity, and reducing power.

Treatment	FRAP (mmol/100 g FW)	DPPH (IC_50_, mg FW/mL)	Reducing power (EC_50_, mg FW/mL)
Attakkari	0.70 ± 0.077^a^	26.07 ± 3.08^b^	33.49 ± 4.10^a^
Bg2907	0.57 ± 0.075	32.66 ± 7.45^b^	38.55 ± 3.52^ab^
Bg406	0.56 ± 0.113	53.66 ± 7.61^a^	40.81 ± 3.65^b^

Values are mean ± standard deviation (*n* = 5). Different superscript letters indicate significant difference at *p* < 0.05.

**Table 2:** The total phenolic content, the total flavonoid content, and the monomeric anthocyanin content.

Sample	TPC (mg TAE/g FW)	TFC (mg TAE/g FW)	Anthocyanin (mg/g FW)
Attakkari	0.900 ± 0.057^a^	6.38 ± 0.82^a^	0.0292 ± 0.009^a^
Bg2907	0.742 ± 0.077^b^	5.59 ± 0.76^a^	0.0247 ± 0.004^a^
Bg406	0.676 ± 0.078^b^	5.36 ± 0.75^a^	0.0202 ± 0.005^a^

Values are mean ± standard deviation (*n* = 5). Different superscript letters indicate significant difference at *p* < 0.05.

**Table 3:** The condensed tannin content.

Sample	CTC (mg CE/g FW)
Attakkari	0.104 ± 0.017^a^
Bg2907	0.099 ± 0.023^a^
Bg406	0.078 ± 0.015^a^

Values are mean ± standard deviation (*n* = 5). Different superscript letters indicate significant difference at *p* < 0.05.

**Table 4:** Correlation (*r*^2^) values among the antioxidant capacity measures of different treatments.

	FRAP (*μ*mol/g FW)	IC_50_ (mg/mL)	EC_50_ (mg/mL)	TPC (TAE) (mg/mL)	TFC (GAE) (mg/mL)
Attakkari					
TPC (TAE)	0.8534	0.7830	0.7136	—	0.7887
TFC (TAE)	0.7146	0.5124	0.9040	—	—
Monomeric anthocyanin (mg/g)	0.887	0.8390	0.9704		0.8011
Condensed tannins (mg CE/g)	0.5503	0.4750	0.3232	0.507	
Bg2907					
TPC (TAE)	0.8124	0.6288	0.7246	—	0.7410
TFC (TAE)	0.7485	0.4575	0.7287	—	—
Monomeric anthocyanin (mg/g)	0.8390	0.8430	0.8380		0.8196
Condensed tannins (mg CE/g)	0.4604	0.4570	0.5439	0.479	
Bg406					
TPC (TAE)	0.8133	0.8170	0.8040	—	0.8761
TFC (TAE)	0.8027	0.7113	0.7610	—	
Monomeric anthocyanin (mg/g)	0.8740	0.8300	0.8380		0.9197
Condensed tannins (mg CE/g)	0.5242	0.5070	0.4036	0.402	
